# Ethnobotanical and phytochemical aspects of the edible herb *Coriandrum sativum* L. 

**DOI:** 10.1111/1750-3841.16085

**Published:** 2022-03-12

**Authors:** Zahra Sobhani, Leila Mohtashami, Mohammad Sadegh Amiri, Mahin Ramezani, Seyed Ahmad Emami, Jesus Simal‐Gandara

**Affiliations:** ^1^ Department of Traditional Pharmacy, School of Pharmacy Mashhad University of Medical Sciences Mashhad Iran; ^2^ Department of Pharmacognosy, School of Pharmacy Mashhad University of Medical Sciences Mashhad Iran; ^3^ Department of Biology Payame Noor University Tehran Iran; ^4^ Nanotechnology Research Center Mashhad University of Medical Sciences Mashhad Iran; ^5^ Pharmaceutical Research Center Mashhad University of Medical Sciences Mashhad Iran; ^6^ Nutrition and Bromatology Group, Department of Analytical Chemistry and Food Science, Faculty of Food Science and Technology University of Vigo—Ourense Campus Ourense Spain

**Keywords:** Apiaceae, coriander, *Coriandrum sativum*, functional foods, traditional medicine

## Abstract

*Coriandrum sativum* (coriander) is an edible herb in the family Apiaceae. The leaves, fruits, and stems of *C. sativum* have long been used as culinary spice due to their favorable odor. Traditional practitioners used this plant for treating different diseases like blepharitis, scabies, aphthous stomatitis, laryngitis, headache, and palpitation. In modern researches, coriander has demonstrated anxiolytic, anticonvulsant, antimigraine, neuroprotective, analgesic, diuretic, hypoglycemic, hypolipidemic, hypotensive, anticancer, and antioxidant activities. Coriander contains a wide range of bioactive phytochemicals among which phenylpropenes, terpenoids, isocoumarins, phytosterols, and fatty acids are the most important. This review provides information about the botanical and ethnobotanical aspects, chemical profile, therapeutic uses in Islamic traditional medicine (ITM), and recent pharmacological studies of coriander effects. The results have shown that coriander and its monoterpenoid compound, linalool, can be considered as potential drug candidates for treating metabolic syndrome and different inflammatory conditions especially neural and CNS diseases.

## 1 INTRODUCTION

Coriander (*Coriandrum sativum* L., family Apiaceae) is an annual herb that has been utilized as a seasoning and therapeutic agent since ancient times (Khan et al., 2014). Coriander has numerous traditional, ethnobotanical, and ethnomedicinal applications and is used for treating different diseases throughout the world. This herb exhibits diaphoretic, diuretic, and carminative properties and is traditionally believed to heal gastrointestinal and respiratory diseases as well as urinary system disorders (Momin et al., 2012; Ugulu et al., 2009). In recent decades, scientists have conducted studies on the chemical components, biological properties, and molecular mechanisms of its biological activities. *Coriandrum sativum* has exerted pharmacological effects like antioxidant, antidiabetic, antimutagenic, anthelminthic, anticonvulsant, anxiolytic, and hepatoprotective (Laribi et al., 2015). These effects are possibly regulated by the potent antioxidant activity of this plant and its main component, linalool. Herein, we have provided information on botanical aspects, ethnobotanical and ethnomedicinal effects, traditional and therapeutic values, and biochemical profile of *C. sativum*.

## 2 TAXONOMY AND BOTANICAL PROFILE OF *C. SATIVUM*

The genus *Coriandrum* is one of the most important genera in the Apiaceae family. The name *Coriandrum* means fetid, which is derived from “koros,” related to the disagreeable fetid smell of the leaves (Tsagkli et al., 2012). This genus is represented by two different species including the cultivated plant *C. sativum* and the wild species *C. tordylium* (Fenzl) Bornm.

*Coriandrum sativum* is a herblike plant that originally came from the Mediterranean region; however, it is being broadly cultivated in Asia, Central Europe, and North Africa for many purposes (Laribi et al., 2015). It is a hairless herb and grows up to a height of about 50 cm. The leaves are diverse in form, being widely obovate at the base of the plant and higher on the flowering stems, they are slender and feathery. The flowers, white or very light pink, are held in delicate umbels. The fruit is a dry globular schizocarp, commonly known as coriander seeds (Omidbaigi, 1997). This valuable species is gaining importance globally and is extensively used by various cultures as a condiment and medicinal plant.

## 3 ETHNOBOTANICAL AND ETHNOMEDICINAL USES

As an edible plant, *C. sativum* has a long tradition and is one of the world's oldest seasoning varieties, dating back to about 1550 bc (Coşkuner & Karababa, 2007). The most frequent traditional applications of *C. sativum* seem to be in the management of gastrointestinal diseases, breathing complications, rheumatism, abdominal complaints, and helminthic disorders throughout the world. Most sections of coriander are safe to eat, but the most common parts used in cooking are leaves and fruits (Momin et al., 2012). Many foods such as fish, beef, and bakery and confectionery products are often flavored using coriander fruits. Indigenous communities assume that using coriander can increase male potency and boost awareness and memory (Khajoei Nasab & Khosravi, 2014). There are remarkable reports on the ethnomedicinal applications of coriander in European traditional medicine. In Turkey, fruits have been recognized as an appetizer, digestive, and carminative (Ugulu et al., 2009). An infusion of the aerial parts is considered very useful in treating abdominal pain (Bulut et al., 2017). In Germany, leaves and fruits (Koriander) are prescribed to ameliorate digestive disorders (Pieroni & Gray, 2008). In Greece, it is used as appetizer, aphrodisiac, carminative, stimulant, and spasmolytic and also for managing dyspepsia, stomach disorders, common cold, and rheumatism (Hanlidou et al., 2004). The leaves and fruits are used in the United Kingdom to treat rheumatism and intestinal disorders, namely flatulence and bloating (Sandhu & Heinrich, 2005). The leaves and young stems are taken for food flavoring in Cyprus (Ciftcioglu, 2015) and the aerial parts (Coentro) are used as condiment in Portugal (Camejo‐Rodrigues et al., 2003).

In Iran, it is known as “Geshniz” and the aerial parts are taken as carminative, calmative, antiseptic, and appetizer (Emami et al., 2012). In Korea, it is believed to be efficacious in the treatment of genitourinary system disorders (Kim & Song, 2011). In Pakistan, it is known as Dhanial and is effective for ameliorating respiratory problems, asthma, cough, bronchitis (Kayani et al., 2014), headache, poor eye sight, low‐grade persistent fever, early ejaculation (Ullah et al., 2014), and hyperlipidemia (Hussain et al., 2018). In Indonesia, the fruits are used for palliating rheumatism and treating syphilis (Silalahi et al., 2015). In the Indian traditional medicine, it is considered as antispasmodic, stimulant, stomachic, carminative, diuretic, and anthelminthic (Sivasankari et al., 2014). In Nepal, the leaf paste is prescribed externally on allergic inflammation and is used orally for treating stomachache (Singh et al., 2012). In Iraq, it is found to be beneficial as a male aphrodisiac, anthelminthic, and carminative (Mati & de Boer, 2011).

In Brazil, the fruits of coriander (Coentro) are used for healing colic (Cartaxo et al., 2010). Coriander is known as a good remedy for catarrh in Cuba (Cano & Volpato, 2004); tonic agent in Guatemala (Girón et al., 1991); diuretic, aperitif, digestive, and antispasmodic in Argentina (Pochettino et al., 2012), and a flavoring agent in Colombia (Rosero‐Toro et al., 2018). Moroccan traditional medicine practitioners use *C. sativum* (Kasbour) to treat bladder ailments, gastric and intestinal pains, muscular and rheumatic pains, insomnia, and diarrhea (Abouri et al., 2012). Coriander is consumed for treating dizziness in Egypt (AbouZid & Mohamed, 2011), foot pain in Sudan (Issa et al., 2018), and used as a spice in Ethiopia (Fenetahun & Eshetu, 2017).

## 4 BIOACTIVE CONSTITUENTS

Numerous studies on the chemical profile of *C. sativum* have indicated that the most important known chemical compounds are phenylpropenes, monoterpenoids, sesquiterpenoids, diterpenoids, phytosterols, fatty acids, isocoumarins, fatty aldehydes, and fatty alcohols (Table 1).

**TABLE 1:** Natural compounds identified in different preparations of *C. sativum*

Name	Structure	Plant part	Preparation	References
Phenylpropenes				
Anethole	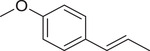	Seed	Essential oil	(Anwar et al., 2011)
		Fruit		(Marichali et al., 2014; Msaada et al., 2009)
Apiol	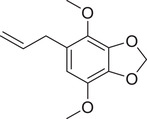	Seed	Essential oil	(Anwar et al., 2011)
Estragole	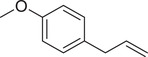	Seed	Essential oil	(Anwar et al., 2011)
Eugenol	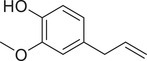	Fruit	Essential oil	(Marichali et al., 2014; Msaada et al., 2009)
Eugenyl acetate	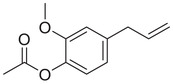	Fruit	Essential oil	(Marichali et al., 2014; Msaada et al., 2009)
Monoterpenoids				
α‐Pinene	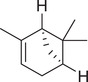	Seed	Essential oil	(Anwar et al., 2011; Orav et al., 2011; Zoubiri & Baaliouamer, 2010)
		Fruit		(Marichali et al., 2014; Msaada et al., 2009; Neffati et al., 2011)
α‐Thujene	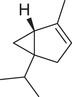	Fruit	Essential oil	(Marichali et al., 2014; Msaada et al., 2009; Neffati et al., 2011)
		Seed		(Anwar et al., 2011)
Camphene	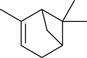	Seed	Essential oil	(Anwar et al., 2011; Orav et al., 2011; Zoubiri & Baaliouamer, 2010)
		Fruit		(Neffati et al., 2011)
Sabinene	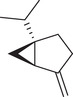	Seed	Essential oil	(Anwar et al., 2011; Orav et al., 2011)
		Fruit		(Marichali et al., 2014; Msaada et al., 2009)
β‐Pinene	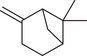	Seed	Essential oil	(Anwar et al., 2011; Orav et al., 2011; Zoubiri & Baaliouamer, 2010)
		Fruit		(Marichali et al., 2014; Msaada et al., 2009)
β‐Myrcene	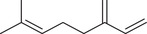	Seed	Essential oil	(Anwar et al., 2011; Orav et al., 2011; Zoubiri & Baaliouamer, 2010)
Δ^3^‐Carene	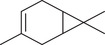	Fruit	Essential oil	(Marichali et al., 2014; Msaada et al., 2009)
*p*‐Cymene	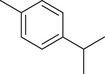	Seed	Essential oil	(Anwar et al., 2011; Orav et al., 2011; Zoubiri & Baaliouamer, 2010)
		Fruit		(Msaada et al., 2009; Neffati et al., 2011)
*p*‐Cymen‐8‐ol	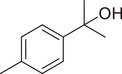	Fruit	Essential oil	(Marichali et al., 2014; Msaada et al., 2009; Neffati et al., 2011)
Phellandrene	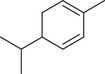	Seed	Essential oil	(Zoubiri & Baaliouamer, 2010)
Menthol	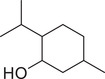	Fruit	Essential oil	(Marichali et al., 2014; Msaada et al., 2009)
Carvone	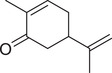	Seed	Essential oil	(Orav et al., 2011)
		Fruit		(Marichali et al., 2014; Msaada et al., 2009)
*p*‐Mentha‐1,4‐dien‐7‐ol	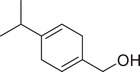	Seed	Essential oil	(Zoubiri & Baaliouamer, 2010)
*p*‐Mentha‐1,8‐diene	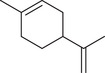	Seed	Essential oil	(Zoubiri & Baaliouamer, 2010)
Menthadien‐1‐ol	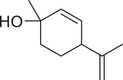	Seed	Essential oil	(Anwar et al., 2011)
Borneol	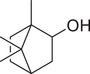	Seed	Essential oil	(Anwar et al., 2011; Zoubiri & Baaliouamer, 2010)
		Fruit		(Marichali et al., 2014; Msaada et al., 2009; Neffati et al., 2011)
Thymol	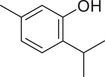	Seed	Essential oil	(Anwar et al., 2011)
		Fruit		(Marichali et al., 2014; Msaada et al., 2009)
Carvacrol	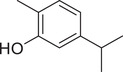	Fruit	Essential oil	(Msaada et al., 2009)
D‐Limonene	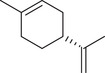	Seed	Essential oil	(Anwar et al., 2011)
Limonene	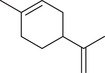	Seed	Essential oil	(Orav et al., 2011; Zoubiri & Baaliouamer, 2010)
		Fruit		(Marichali et al., 2014; Msaada et al., 2009; Neffati et al., 2011)
α‐Terpinene	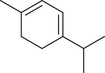	Fruit	Essential oil	(Marichali et al., 2014; Msaada et al., 2009)
γ‐Terpinene	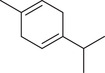	Seed	Essential oil	(Anwar et al., 2011; Orav et al., 2011)
		Fruit		(Marichali et al., 2014; Msaada et al., 2009; Neffati et al., 2011)
Terpinolene	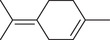	Seed	Essential oil	(Anwar et al., 2011)
		Fruit		(Marichali et al., 2014; Msaada et al., 2009)
Terpinene‐4‐ol	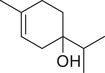	Seed	Essential oil	(Anwar et al., 2011; Orav et al., 2011; Zoubiri & Baaliouamer, 2010)
		Fruit		(Marichali et al., 2014; Msaada et al., 2009; Neffati et al., 2011)
α‐Terpineol	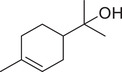	Seed	Essential oil	(Anwar et al., 2011; Orav et al., 2011)
		Fruit		(Marichali et al., 2014; Msaada et al., 2009; Neffati et al., 2011)
1,8‐Cineole	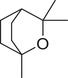	Fruit	Essential oil	(Marichali et al., 2014; Msaada et al., 2009)
(Z)‐β‐Ocimene	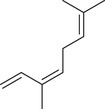	Fruit	Essential oil	(Marichali et al., 2014; Msaada et al., 2009)
Citronellal	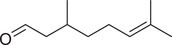	Seed	Essential oil	(Anwar et al., 2011)
β‐Citronellol	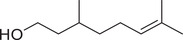	Seed	Essential oil	(Anwar et al., 2011)
		Fruit		(Marichali et al., 2014; Msaada et al., 2009)
Linalool	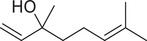	Seed	Essential oil	(Anwar et al., 2011; Orav et al., 2011; Zoubiri & Baaliouamer, 2010)
		Fruit		(Marichali et al., 2014; Msaada et al., 2009; Neffati et al., 2011)
Linalyl acetate	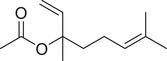	Seed	Essential oil	(Orav et al., 2011; Zoubiri & Baaliouamer, 2010)
*cis*‐Linalool oxide (furanoid)	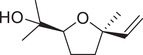	Fruit	Essential oil	(Marichali et al., 2014; Msaada et al., 2009)
*trans*‐Linalool oxide	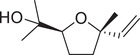	Fruit	Essential oil	(Msaada et al., 2009)
Camphor	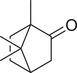	Seed	Essential oil	(Anwar et al., 2011; Orav et al., 2011; Zoubiri & Baaliouamer, 2010)
		Fruit		(Marichali et al., 2014; Msaada et al., 2009; Neffati et al., 2011)
Geraniol	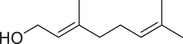	Seed	Essential oil	(Orav et al., 2011)
		Fruit		(Marichali et al., 2014; Msaada et al., 2009; Neffati et al., 2011)
Geranial	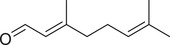	Seed	Essential oil	(Orav et al., 2011)
		Fruit		(Marichali et al., 2014; Msaada et al., 2009)
Geranyl acetate	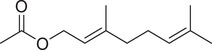	Seed	Essential oil	(Orav et al., 2011; Zoubiri & Baaliouamer, 2010)
		Fruit		(Marichali et al., 2014; Msaada et al., 2009; Neffati et al., 2011)
Nerol	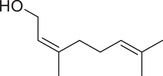	Fruit	Essential oil	(Marichali et al., 2014; Msaada et al., 2009; Neffati et al., 2011)
Neral	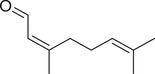	Fruit	Essential oil	(Marichali et al., 2014; Msaada et al., 2009)
Neryl acetate	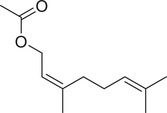	Seed	Essential oil	(Zoubiri & Baaliouamer, 2010)
		Fruit		(Msaada et al., 2009; Neffati et al., 2011)
Lavandulol	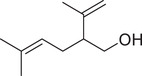	Seed	Essential oil	(Zoubiri & Baaliouamer, 2010)
Cuminaldehyde	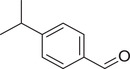	Seed	Essential oil	(Anwar et al., 2011)
*cis*‐Dihydrocarvone	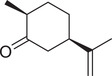	Seed	Essential oil	(Zoubiri & Baaliouamer, 2010)
		Fruit		(Marichali et al., 2014; Msaada et al., 2009)
Sesquiterpenoids				
*cis*‐Nerolidol	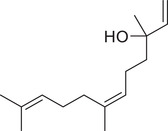	Seed	Essential oil	(Anwar et al., 2011)
δ‐Elemene	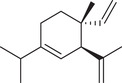	Fruit	Essential oil	(Marichali et al., 2014; Msaada et al., 2009)
α‐Elemol	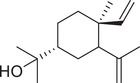	Seed	Essential oil	(Anwar et al., 2011)
β‐Caryophyllene	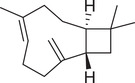	Seed	Essential oil	(Anwar et al., 2011)
		Fruit		(Marichali et al., 2014; Msaada et al., 2009; Neffati et al., 2011)
α‐Cubebene	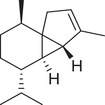	Seed	Essential oil	(Anwar et al., 2011)
δ‐Cadinene	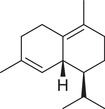	Seed	Essential oil	(Anwar et al., 2011)
α‐Humulene	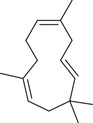	Fruit	Essential oil	(Marichali et al., 2014; Msaada et al., 2009; Neffati et al., 2011)
		Seed		(Orav et al., 2011)
Germacrene D	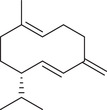	Fruit	Essential oil	(Marichali et al., 2014; Msaada et al., 2009)
Santalol	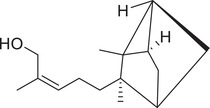	Seed	Essential oil	(Anwar et al., 2011)
Tocopherols				
α‐Tocopherol	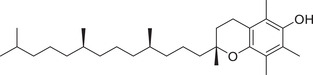	Whole fruit	Hexane extract	(Sriti et al., 2010)
		Seed		
		Pericarp		
α‐Tocotrienol	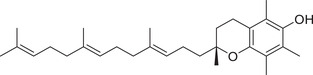	Whole fruit	Hexane extract	(Sriti et al., 2010)
		Seed		
β‐Tocopherol	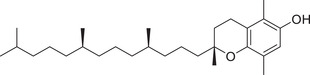	Whole fruit	Hexane extract	(Sriti et al., 2010)
		Pericarp		
γ‐Tocopherol	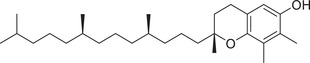	Whole fruit	Hexane extract	(Sriti et al., 2010)
		Seed		
		Pericarp		
γ‐Tocotrienol	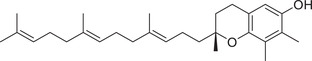	Whole fruit	Hexane extract	(Sriti et al., 2010)
		Seed		
		Pericarp		
δ‐Tocopherol	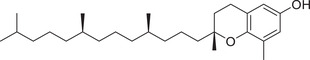	Seed	Hexane extract	(Sriti et al., 2010)
		Pericarp		
δ‐Tocotrienol	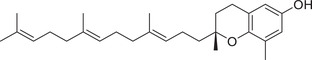	Whole fruit	Hexane extract	(Sriti et al., 2010)
		Seed		
Phytosterols				
Cholesterol	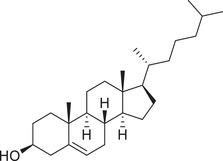	Whole fruit	Hexane extract	(Sriti et al., 2009)
		Seed		
		Pericarp		
Campesterol	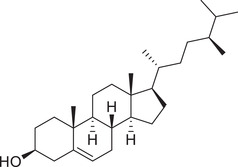	Whole fruit	Hexane extract	(Sriti et al., 2009)
		Seed		
		Pericarp		
Stigmasterol	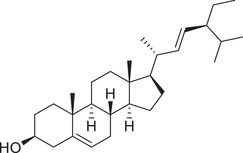	Whole fruit	Hexane extract	(Sriti et al., 2009)
		Seed		
		Pericarp		
β‐Sitosterol	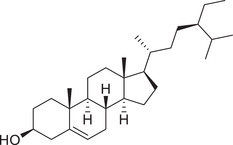	Whole fruit	Hexane extract	(Sriti et al., 2009)
		Seed		
		Pericarp		
Δ^5^‐Avenasterol	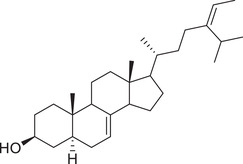	Whole fruit	Hexane extract	(Sriti et al., 2009)
		Seed		
		Pericarp		
Δ^5^‐24‐Stigmastadienol	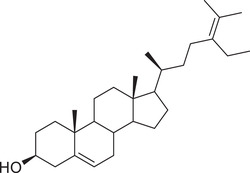	Whole fruit	Hexane extract	(Sriti et al., 2009)
		Seed		
		Pericarp		
Δ^7^‐Stigmasterol	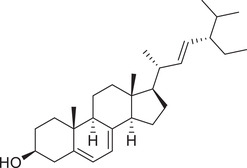	Whole fruit	Hexane extract	(Sriti et al., 2009)
		Seed		
		Pericarp		
Δ^7^‐Avenasterol	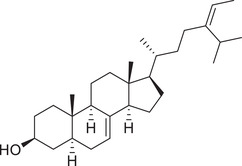	Whole fruit	Hexane extract	(Sriti et al., 2009)
		Seed		
		Pericarp		
Fatty acids				
Myristic acid	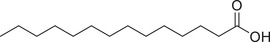	Seed	Chloroform‐Methanol (2:1) extract	(Marichali et al., 2014)
		Leaf		
		Stem		
		Root		
Pentadecanoic acid	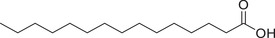	Seed	Chloroform‐Methanol (2:1) extract	(Marichali et al., 2014)
		Leaf		
		Stem		
		Root		
Palmitic acid	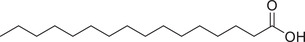	Seed	Chloroform‐Methanol (2:1) extract	(Marichali et al., 2014)
		Leaf		
		Stem		
		Root		
		Whole fruit	Hexane extract	(Sriti et al., 2009)
		Seed		
		Pericarp		
Tridecanoic acid	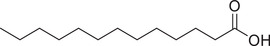	Seed	Essential oil	(Anwar et al., 2011)
Hexadecanoic acid	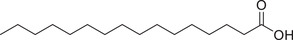	Seed	Essential oil	(Anwar et al., 2011)
Tetradecanoic acid	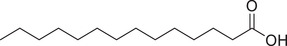	Seed	Essential oil	(Zoubiri & Baaliouamer, 2010)
Arachidic acid	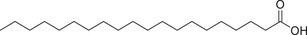	Seed	Chloroform‐Methanol (2:1) extract	(Marichali et al., 2014)
		Leaf		
		Stem		
		Root		
		Whole fruit	Hexane extract	(Sriti et al., 2009)
		Seed		
		Pericarp		
Heptadecanoic acid	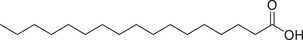	Seed	Chloroform‐Methanol (2:1) extract	(Marichali et al., 2014)
		Leaf		
		Stem		
		Root		
Stearic acid	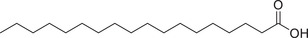	Seed	Chloroform‐Methanol (2:1) extract	(Marichali et al., 2014)
		Leaf		
		Stem		
		Root		
		Whole fruit	Hexane extract	(Sriti et al., 2009)
		Seed		
		Pericarp		
Palmitoleic acid	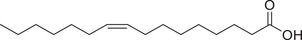	Seed	Chloroform‐Methanol (2:1) extract	(Marichali et al., 2014)
		Leaf		
		Stem		
		Root		
		Whole fruit	Hexane extract	(Sriti et al., 2009)
		Seed		
		Pericarp		
Petroselinic acid	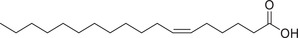	Seed	Chloroform‐Methanol (2:1) extract	(Marichali et al., 2014)
		Whole fruit	Hexane extract	(Sriti et al., 2009)
		Seed		
		Pericarp		
Oleic acid	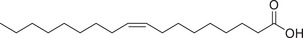	Seed	Chloroform‐Methanol (2:1) extract	(Marichali et al., 2014)
		Leaf		
		Stem		
		Root		
		Whole fruit	Hexane extract	(Sriti et al., 2009)
		Seed		
		Pericarp		
Linoleic acid	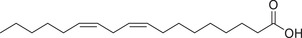	Seed	Chloroform‐Methanol (2:1) extract	(Marichali et al., 2014)
		Leaf		
		Stem		
		Root		
		Whole fruit	Hexane extract	(Sriti et al., 2009)
		Seed		
		Pericarp		
α‐Linolenic acid	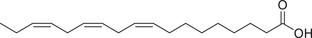	Seed	Chloroform‐Methanol (2:1) extract	(Marichali et al., 2014)
		Leaf		
		Stem		
		Root		
		Whole fruit	Hexane extract	(Sriti et al., 2009)
		Seed		
		Pericarp		
Gadoleic acid	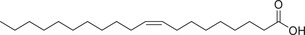	Seed	Chloroform‐Methanol (2:1) extract	(Marichali et al., 2014)
		Leaf		
		Stem		
		Root		
Erucic acid	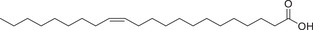	Seed	Chloroform‐Methanol (2:1) extract	(Marichali et al., 2014)
		Leaf		
		Stem		
		Root		
Isocoumarins				
Coriandrone A	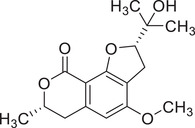	–	–	(Taniguchi et al., 1996)
Coriandrone B	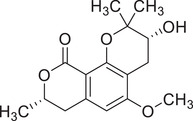	–	–	(Taniguchi et al., 1996)
Coriandrone C	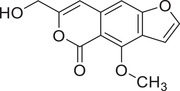	Whole plant	Methanol extract	(Taniguchi et al., 1996)
Coriandrone D	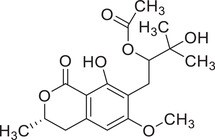	Whole plant	Methanol extract	(Taniguchi et al., 1996)
Coriandrone E	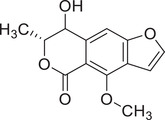	Whole plant	Methanol extract	(Taniguchi et al., 1996)
Coriandrin	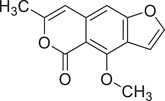	–	–	(Taniguchi et al., 1996)
Dihydrocoriandrin	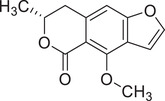	–	–	(Taniguchi et al., 1996)
Fatty aldehydes				
Heptanal	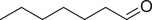	Seed	Essential oil	(Anwar et al., 2011)
		Fruit	Essential oil	(Marichali et al., 2014; Msaada et al., 2009)
Octanal	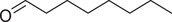	Seed	Essential oil	(Anwar et al., 2011)
		Leaf	Essential oil	(Freires et al., 2014)
Decanal	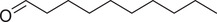	Seed	Essential oil	(Anwar et al., 2011; Zoubiri & Baaliouamer, 2010)
		Leaf	Essential oil	(Freires et al., 2014)
Undecanal	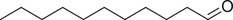	Seed	Essential oil	(Zoubiri & Baaliouamer, 2010)
		Leaf	Essential oil	(Freires et al., 2014)
Dodecanal	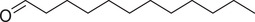	Seed	Essential oil	(Anwar et al., 2011)
		Leaf	Essential oil	(Freires et al., 2014)
Tridecanal	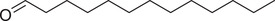	Seed	Essential oil	(Zoubiri & Baaliouamer, 2010)
Tetradecanal	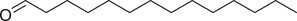	Seed	Essential oil	(Anwar et al., 2011)
2‐Undecenal	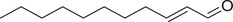	Leaf	Essential oil	(Freires et al., 2014)
*trans*‐4‐Decenal	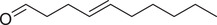	Leaf	Essential oil	(Freires et al., 2014)
*trans*‐2‐Decenal	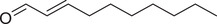	Seed	Essential oil	(Freires et al., 2014; Zoubiri & Baaliouamer, 2010)
		Leaf	Essential oil	(Freires et al., 2014)
*cis*‐2‐Dodecenal	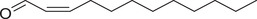	Leaf	Essential oil	(Freires et al., 2014)
2E‐1‐Tridecenal	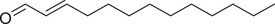	Seed	Essential oil	(Zoubiri & Baaliouamer, 2010)
Fatty alcohols				
1‐Decanol	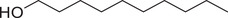	Seed	Essential oil	(Zoubiri & Baaliouamer, 2010)
1‐Undecanol	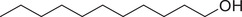	Seed	Essential oil	(Anwar et al., 2011; Zoubiri & Baaliouamer, 2010)
1‐Dodecanol	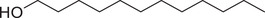	Leaf	Essential oil	(Freires et al., 2014)
3‐Hexen‐1‐ol	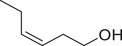	Fruit	Essential oil	(Neffati et al., 2011)
2‐Decen‐1‐ol	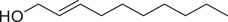	Seed	Essential oil	(Freires et al., 2014; Zoubiri & Baaliouamer, 2010)
		Leaf	Essential oil	(Freires et al., 2014)
*trans*‐2‐Undecen‐1‐ol	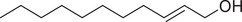	Seed	Essential oil	(Zoubiri & Baaliouamer, 2010)
		Leaf	Essential oil	(Freires et al., 2014)
Fatty esters				
*cis*‐3‐Hexenyl butyrate	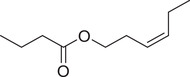	Fruit	Essential oil	(Marichali et al., 2014; Msaada et al., 2009)
Hydrocarbons				
2,6‐Octadiene	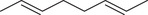	Seed	Essential oil	(Anwar et al., 2011)
Nonadecane	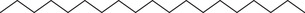	Seed	Essential oil	(Anwar et al., 2011)
Cyclodecane	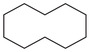	Leaf	Essential oil	(Freires et al., 2014)
Heneicosane	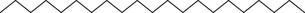	Seed	Essential oil	(Anwar et al., 2011)
Z‐5‐Nonadecene	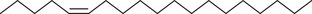	Seed	Essential oil	(Anwar et al., 2011)
Miscellaneous				
2‐Methyl‐3‐phenyl‐propanal	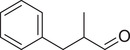	Seed	Essential oil	(Zoubiri & Baaliouamer, 2010)
2‐Pentadecanone	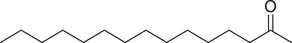	Seed	Essential oil	(Anwar et al., 2011)

## 5 TRADITIONAL USES OF *C. SATIVUM* IN ITM

There are two kinds of coriander in Islamic traditional medicine (ITM) textbooks. One grows in the desert and has rounded leaves and tiny green seeds, while the other flourishes in gardens and has larger leaves and seeds (Ibn Beyṭâr, 2001; Shirâzi, 2014). In ITM, coriander has been shown to be of benefit in treating different diseases that have been categorized subsequently.

### 5.1 Eye diseases

For the treatment of blepharitis and conjunctivitis, a poultice developed from coriander leaves with human milk or dry bread is used. Also, it has been mentioned that using the extract of leaves as an eye drop will inhibit the occurrence of smallpox and measles in this organ (Anṭâki, 2000; Ibn Sinâ, 2016; Jorjâni, 1977; Shirâzi, 2014).

### 5.2 Skin diseases

Topical administration of a mixture from coriander leaves, dried bread, or barley and bean flour is effective for healing erysipelas, scrofula, itching, scabies, herpes, and hot inflammation (bib142Anṭâki, 2000; Ibn Sinâ, 2016; Râzi, 2002; Shirâzi, 1992; Shirâzi, 2014). A combination of lead, coriander leaf extract, and rose oil has been used to prevent the expansion of progressive ulcers (Râzi, 2002; Shirâzi, 2014). Furthermore, topical application of the seeds with olive oil and honey has been prescribed for treating hives and anthrax (Ibn Beyṭâr, 2001; Shirâzi, 2014).

### 5.3 Oral cavity diseases

Coriander leaves are a recommended treatment for aphthous stomatitis, toothache, and gum hemorrhages (Herawi, 1992; Ibn Sinâ, 2016; Shirâzi, 2014).

### 5.4 Respiratory system diseases

It is reported that a gargle prepared from coriander seeds with rose hydrola is a strong remedy for laryngitis (Herawi, 1992). Also, nasal administration of the leaf extract is beneficial for nose bleeding (Ibn Sinâ, 2016). Shirâzi (2014) has stated in his book that the syrup of the leaves is beneficial for ameliorating cough and asthma.

### 5.5 Gastrointestinal diseases

In ITM, the movement of harmful humors from stomach to the head can cause headache. A poultice prepared from the seeds can strengthen the stomach and decrease the mobility of these humors (Qarshi, 2005). In different traditional medicine books, oral consumption of coriander leaves with sugar is believed to be an appetizer and hypnotic, and is prescribed to treat gastritis, vomiting, and nausea (Ibn Sinâ, 2016; Shirâzi, 2014). The seeds are said to be a stomach tonic and a combination of the seeds with *Santalum* and aniseed can ameliorate burping (Anṭâki, 2000; Ibn Beyṭâr, 2001; Ibn Sinâ, 2016). Râzi (2002) noted that consuming coriander seeds with pepper is a useful remedy for vomiting after eating food and can enhance food transit time from stomach.

### 5.6 Cardiovascular diseases

Topical administration of coriander seeds with olive oil and honey can ameliorate varicocele and hot inflammation of testicular vessels (Ansâri Shirâzi, 1992; Ibn Beyṭâr, 2001; Shirâzi, 2014). Traditional scientists prescribed the seeds to treat palpitation (Herawi, 1992; Ibn Sinâ, 2016).

### 5.7 Other diseases

A syrup prepared from coriander leaves is effective for ameliorating dizziness and tinnitus (Ansâri Shirâzi, 1992; Anṭâki, 2000; Shirâzi, 2014). Coriander seeds are protective against obsessive‐compulsive disorder (Herawi, 1992; Ibn Sinâ, 2016) and from the perspective of Rhazes, coriander seeds with concentrated grape juice increase semen content and treat parasitic worm infections (Anṭâki, 2000; Ghasâni, 1990; Râzi, 2002).

High dose consumption of coriander seeds can cause forgetfulness, mental disorders, dizziness, hoarseness, reduced semen content, and weakness in sexual power (Ansâri Shirâzi, 1992; Shirâzi, 2014).

## 6 PHARMACOLOGICAL ASPECTS

### 6.1 Antioxidant effect

One of the primary causes of metabolic syndrome, Alzheimer's disease, Parkinson's disease, cardiovascular diseases, stroke, chronic kidney disease, and chronic pulmonary obstructive disease is oxidative stress (Barnham et al., 2004; Dhalla et al., 2000; Khansari et al., 2009; Perera & Handuwalage, 2015; Rahman, 2005; Small et al., 2012).

It has been shown that oral administration of ethanol extract obtained from coriander leaves caused a significant reduction in creatinine, serum urea, and blood urea nitrogen levels in rats with nephrotoxicity (Lakhera et al., 2015). In the same manner, flavonoids of the aqueous extract of coriander seeds exhibited a suppressive activity to oxidative injury in animals with renal and liver lead toxicity. Oral consumption of these extracts increased superoxide dismutase (SOD), catalase (CAT), and glutathione (GSH) levels and reduced lipid peroxidation (Samojlik et al., 2010). Methanol extract of coriander fruits has shown a significant 1,1‐diphenyl‐2‐picrylhydrazyl (DPPH) radical scavenging effect indicating the potential of coriander fruits as a natural source of antioxidant compounds that can be used in food industry (Msaada et al., 2017; Sultana et al., 2010). Also, the hydro‐alcohol extract of coriander leaves could reduce lipid peroxidation and DNA damage (Harsha & Anilakumar, 2014).

### 6.2 Antimicrobial and anthelminthic effects

One of the most recorded biological actions of *C. sativum* is the antimicrobial function of the leaves, seeds, and essential oils (Silva & Domingues, 2017; Sundar et al., 2016). The essential oil had a notable inhibition zone against gram‐positive (*Staphylococcus aureus* and *Bacillus* spp.) and gram‐negative (*Escherichia coli*, *Klebsiella pneumonia*, *Pseudomonas aeruginosa*, *Proteus mirabilis*, and *Salmonella typhi*) bacteria. Also, it could strongly inhibit biofilm development of *S. aureus*, *E. coli* (Mohammadi Bazargani & Rohloff, 2016), *Stenotropomonas maltophilia* (Kačániová et al., 2020) and *Campylobacter jejuni* and *C. coli*. Therefore, essential oil and linalool can be considered as natural additives or preservatives in the food industry that can increase food shelf‐life and safety (Duarte et al., 2016). Hexane and chloroform extracts of coriander fruits have demonstrated strong inhibitory activity against biofilm formation of *Salmonella enterica*, *E. coli*, *P. aeruginosa*, and especially *S. aureus* (Molina et al., 2020). The growth of *B. subtilis* and *E. coli* could be suppressed by methanol and aqueous extracts of the leaves and stems. It is important to mention that methanol extract of coriander leaves has a stronger inhibitory effect in comparison to other extracts, which may possibly be due to its higher total phenol content (Wong & Kitts, 2006). Coriander essential oil has shown a synergistic activity with six separate antibacterial drugs: ciprofloxacin, chloramphenicol, gentamicin, cefoperazone, tetracycline, and piperacillin. Also, an interaction between coriander essential oil and chloramphenicol, ciprofloxacin, gentamicin, and tetracycline against *Acinetobacter baumannii* was observed, which may be an indication of potential efficacy of essential oil (Duarte et al., 2012).

The essential oil of coriander seeds demonstrated antifungal action against *Candida albicans* and the essential oil of coriander leaves could inhibit *Candida* species (Begnami et al., 2010; Lo Cantore et al., 2004; Msaada et al., 2007). The essential oil of fruits could inhibit *Microsporum canis* strains (Soares et al., 2012) and the essential oil of coriander seeds was efficient against *Fusarium oxysporum*, *Curvularia palliscens*, *F. moniliforme*, *Aspergillus terreus* (Singh et al., 2006) and insects of grains, *Sitophilus granarius* (Zoubiri & Baaliouamer, 2010). Ethanol extract of coriander seeds could destroy adult tapeworm, *Hymenolepis nana*. This effect was increased dose‐dependently as higher doses could eliminate the worm in a shorter period of time (Hosseinzadeh et al., 2016).

### 6.3 Anticancer effect

Phenolic compounds possess antiproliferative activity via downregulating oxidative stress, DNA injury, and controlling abnormal cell growth (Cai et al., 2004; Hashim et al., 2005; Tang et al., 2013). Colorectal cancer or bowel cancer is the third‐most prevalent cancer in both sexes globally (Munkholm, 2003). The influence of ethanol extract of coriander leaves on HT‐29 colon cancer cells revealed a dose‐dependent decrease in cell viability that may be related to polyphenolic compounds (Nithya & Sumalatha, 2014). Linalool is one of the major components present in coriander essential oil. This monoterpenoid compound can mildly prevent cell proliferation. Subtoxic linalool amounts could upregulate doxorubicine (DOX)‐induced cytotoxicity and pro‐apoptotic influence on breast cancer cell lines, MCF‐7, and multidrug‐resistant MCF‐7. This effect may be due to the ability of linalool in enhancing DOX accumulation (Ravizza et al., 2008). Treating HCT‐116 colon cancer cells with 250 µM linalool has resulted in chromatin fragmentation and cell shrinkage, which can demonstrate apoptosis and cell death. Also, oral administration of linalool to a cancer xenografted mouse model decreased weight and size of subcutaneous tumors (Iwasaki et al., 2016).

### 6.4 Anxiolytic effect

Coriander is used to treat insomnia, headache, and anxiety in traditional and folk medicine (Duke et al., 2002). The high affinity of coriander flavonoids such as quercetin and isoquercetin to central benzodiazepine receptors may result in anxiolytic effect. The hydro‐alcohol extract of aerial parts and its ethyl acetate and butanol fractions showed hypnotic effects in mouse model by increasing sleeping time (Rakhshandeh et al., 2012). Intraperitoneal administration of aqueous extract of coriander seeds has shown a significant anxiolytic effect in mice. Also, essential oil of coriander was linked to gamma γ‐aminobutyric acid A (γ‐GABA_A_) receptor complex and exerted antidepressant and anxiolytic properties (Emamghoreishi et al., 2005).

Numerous studies focus on the effects of linalool on central nervous system. This compound has been shown to reduce locomotor activity without changing coordination and body temperature (Linck et al., 2009). Linalool inhalation enhanced social interactions, reduced offensive actions (Souto‐Maior et al., 2011), and exerted anxiolytic effect (Linck et al., 2010). Intracerebrovascular administration of linalool and coriander essential oil to neonatal chicks demonstrated that linalool is responsible for the sedative activity of coriander (Gastón et al., 2016). This compound, like benzodiazepines, enhanced GABA effect on its receptor, which resulted in anticonvulsant, anxiolytic, sedative, hypnotic, and muscle‐relaxant effects (Elisabetsky et al., 1999). Some studies relate the anxiolytic effect of linalool to mediation of glutamatergics and nicotinic acid (Elisabetsky et al., 1995; Re et al., 2000). Further, decreased secretion of monoamines like norepinephrine, serotonin, and dopamine has occurred in the hippocampus and cortex of linalool‐treated mice (Cheng et al., 2015). These mice showed an increased dopamine level in the stratum; thus, linalool may be beneficial for treating Parkinson's disease as well (Cheng et al., 2015).

### 6.5 Antiseizure effect

Epilepsy is a very common neurological disease where oxidative stress plays an important role in its pathogenesis (Shin et al., 2011). Epilepsy is defined as frequent seizures that can raise the content of oxygen free radicals in the brain (Sudha et al., 2001), leading to cognitive and psychiatric problems in patients (Reilly et al., 2011). As a result, antioxidant compounds possess a vital role for managing seizures (Aguiar et al., 2012). Coriander extracts decrease malondialdehyde (MDA) level and increase total thiol content, thus, improving the antioxidant capacity of the brain. Various extracts of coriander aerial parts can remarkably prolong seizure latencies (Anaeigoudari et al., 2016; Karami et al., 2015). This effect may be attributed to linalool as the main compound, which works by inhibiting glutamate attachment in the rat cortex (Anaeigoudari et al., 2016). The mechanisms of linalool effects on the central nervous system are similar to those of conventional antianxiety and anticonvulsant drugs, namely GABA_A_/chloride channel receptor activation and voltage‐gated sodium and calcium channel suppression (White et al., 2007). An in vivo study on the epilepsy model exhibited that minimum‐dose linalool enhanced the action potential threshold, extended poststimulus period, and reduced action potential rising phase. In contrast, high‐dose administration of linalool stimulated neurons and increased epileptogenic activity (Vatanparast et al., 2017). In seizures and epilepsies, the concentration of glutamate, the main excitatory neurotransmitter in the brain, increases. Linalool can block the glutamatergic transmission by inhibiting L‐[3H] glutamate binding, preventing quinolinic acid‐induced convulsions, delaying the onset of N‐methyl‐d‐aspartic acid (NMDA)‐induced seizure, and reducing cyclic adenosine monophosphate (cAMP) level (Elisabetsky et al., 1999).

### 6.6 Antimigraine effect

Migraine is characterized by frequent headaches ranging from moderate to severe. Headaches typically affect half of the head, are naturally pulsating, and last from a few hours to 3 days (World Health Organization, 2016). Migraine pain is suggested to be in association with an interaction between neural pathways that caused neuronal hyperexcitability and dilation of intracranial vessels (Akerman et al., 2017). Another leading mechanism proposed for migraine is the release of pro‐inflammatory mediators (Aurora et al., 2011). Linalool has anti‐inflammatory and analgesic effects, leading to migraine relief (Delavar Kasmaei et al., 2016; Peana et al., 2003). Linalool can be an antagonist of the glutamatergic receptor; thus, it can inhibit glutamate transmission and ameliorate migraine (Batista et al., 2008).

The transient receptor potential M8 (TRPM8) channel is a nonselective ion channel that is sensitive to cold temperature and cooling factors such as menthol (Dussor & Cao, 2016; Weyer & Lehto, 2017). The level of TRPM8 expression will increase in migraine and the pain neuronal circuitry, such as trigeminal and dorsal root ganglia. The expression of this gene is influential in the pathophysiology of migraine (Akerman et al., 2017; Ligthart et al., 2011) and linalool has shown to be an antagonist of this channel (Behrendt et al., 2004).

### 6.7 Neuroprotective effect

The brain is susceptible to oxidative stress injury due to its physiological and biochemical properties (Uttara et al., 2009). There is a straight relationship between memory and oxidative stress because increased lipid peroxidation and MDA level has led to working memory errors. Coriander has improved memory in rats who have inhaled its essential oil and this effect was attributed to the antioxidant properties of coriander. This is so because oxidative markers like MDA, SOD, and H_2_O_2_ decreased and glutathione peroxidase (GPX) activity increased in the hippocampus of coriander‐treated animals. Furthermore, lactate dehydrogenase (LDH) activity, amyloid β‐protein level, and DNA cleavage pattern in hippocampus were reduced. Linalool as the major compound of essential oil was believed to be responsible for most of these effects (Cioanca et al., 2013). Similarly, inhalation of coriander oil is effectual in treating anxiety and depression‐like actions in a rat model of Alzheimer's disease (Cioanca et al., 2014). It should be noted that inflammation is a crucial factor in the occurrence of Alzheimer's, and coriander has indicated a remarkable anti‐inflammatory activity (Huo et al., 2013; Ma et al., 2015). Oral administration of linalool to triple a transgenic mice model of Alzheimer's disease decreased P38 mitogen‐activated protein kinase (p38 MAPK), cyclooxygenase‐2 (COX‐2), nitric oxide synthase 2 (NOS2), and interleukin 1 beta (IL‐1β) that resulted in anti‐inflammatory effects (Sabogal‐Guáqueta et al., 2016).

In addition to neurological diseases, neurotoxicity due to acrylamide exposure is another major concern. Acrylamide exposure is more common in some occupations, but the greatest concern is related to the presence of acrylamide in foods. This can indisputably increase the occurrence of different diseases (Capuano & Fogliano, 2011; Dybing et al., 2005). Acrylamide toxicity has enhanced lipid peroxidation leading to a decrease in the antioxidant capacity of the brain (Zhu et al., 2008). This can cause neuronal apoptosis to evolve by mediating caspase‐3 activity (Sumizawa & Igisu, 2007). Mehri et al. reported that linalool administration 3 days before acrylamide‐induced neurotoxicity could decrease lipid peroxidation and MDA and increase glutathione in treated Wistar rats. They suggested that the antioxidant property of linalool is a leading mechanism for its neuroprotective effect. However, linalool administration after acrylamide exposure could not prevent neurotoxicity (Mehri et al., 2015).

### 6.8 Analgesic effect

Different extracts of coriander aerial parts have shown analgesic effects. Among them, chloroform and ethanol extracts were more effective (Kazempor et al., 2015). Intraperitoneal administration of ethanol extract of coriander seeds at 200 mg/kg has resulted in 50% pain inhibition in mice (Pathan et al., 2011). The pain‐relieving activity of aqueous extract of coriander seeds was assessed using hot plate, tail flicking, and formalin methods. The aqueous extract was found to exhibit potent analgesic activity in a dose‐dependent manner (Taherian et al., 2012). The presence of various chemicals such as polyphenols and linalool may be responsible for the analgesic action of coriander (de Campos Buzzi et al., 2009; Kaur et al., 2005). The probable mechanism for the antinociceptive effect of these compounds may be due to the involvement of GABAergic transmission, which interacts with central benzodiazepine receptors (Lau & Vaughan, 2014; Youdim et al., 2004). It may also be related to the activation of opiate system because pretreatment with naloxone (a nonselective opiate antagonist) decreased the pain‐relieving effect of coriander (Kazempor et al., 2015; Taherian et al., 2012). Multiple analgesic pathways of linalool consist of glutamatergic (ionotropic glutamate receptors), muscarinic (M_2_ receptors), opioid, adenosinergic (A_1_ and A_2_ receptors), and dopaminergic (D_2_ receptors) systems (Batista et al., 2010; Venâncio et al., 2011). Linalool blocked NMDA and other ionotropic glutamate receptors in the glutamate pathway and therefore exerted a modulatory effect on chronic pain (Batista et al., 2008; Beirith et al., 2002; Chizh et al., 2001). Systemic prescription of linalool could decrease severe thermal hyperalgesia reaction in the animal model by inhibiting NMDA receptors (Peana et al., 2004). This compound has led to analgesia in chronic pain states like persistent inflammation and neuropathic ailments by inhibiting NMDA receptors, glutamate uptake, and nitric oxide (NO) formation (Batista et al., 2010). Linalool was able to adjust the nicotinic receptor‐ion channel kinetics at neuromuscular junctions, which changed the secretion of acetylcholine. This mechanism was the underlying reason for the anesthetic‐like effect of linalool (Re et al., 2000).

### 6.9 Metabolic syndrome

Metabolic syndrome refers to a set of conditions that include high blood pressure, elevated levels of blood insulin, excess accumulated fat around the abdomen, and elevated levels of blood lipids. The mentioned criteria lead to an increased risk of heart disease, stroke, and diabetes. Nowadays, metabolic syndrome is a global concern due to its association with socio‐economic status (Després & Lemieux, 2006; Grundy, 2008). The mechanisms involved in the therapeutic effects of coriander for treating metabolic syndrome are expressed subsequently.

#### 6.9.1 Hypolipidemic effect

**FIGURE 1:**
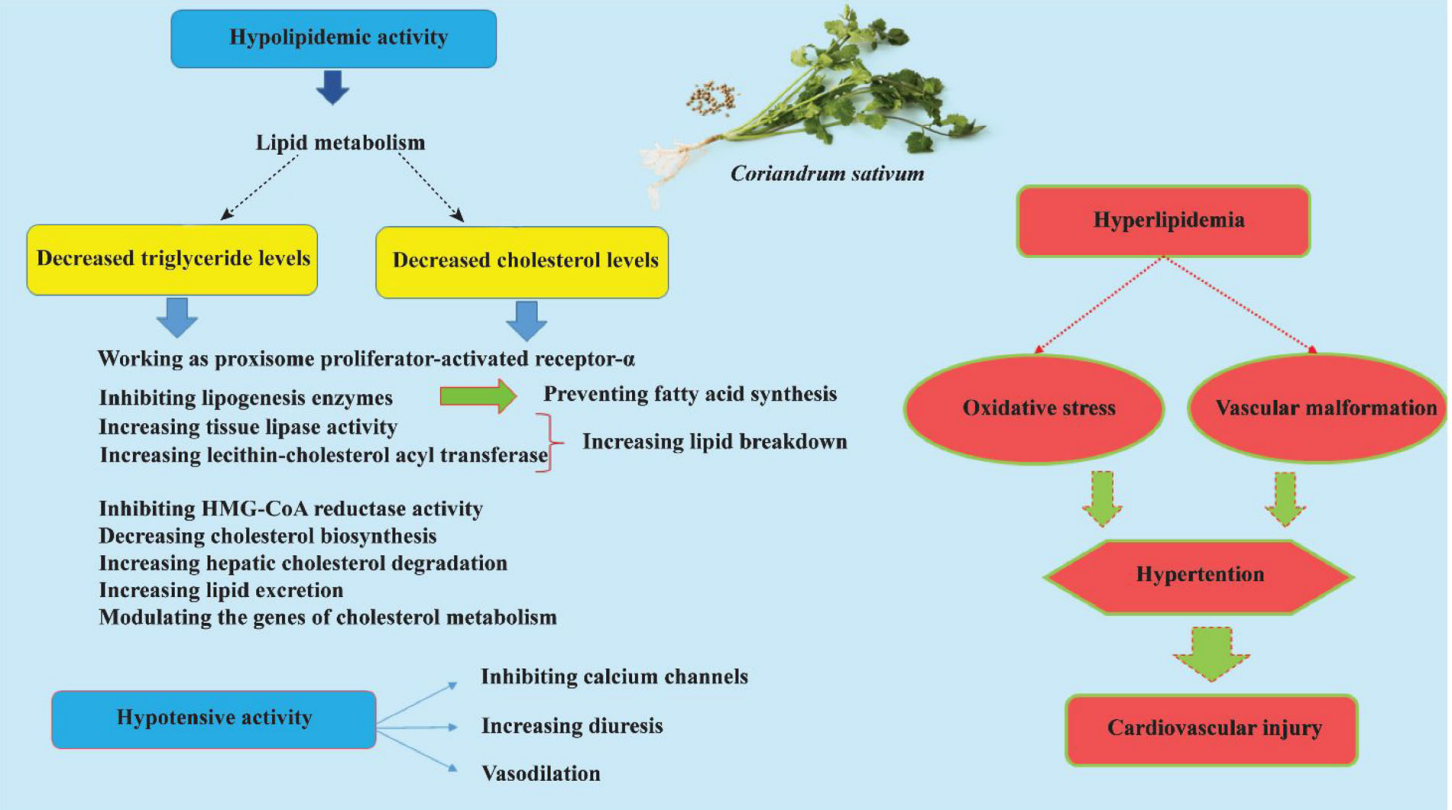
Schematic description of hypolipidemic and hypotensive mechanisms of *C. sativum*

Dyslipidemia management plays a vital role in preventing cardiovascular diseases, especially in patients with diabetes (Miller, 2009). The results of many in vivo studies have indicated that *C. sativum* affects the lipid profile. Coriander can modulate different enzymes of lipid metabolism pathways and decrease triglyceride (TG) and cholesterol levels. As an example, the activity of 3‐hydroxy‐3‐methyl‐glutaryl‐coenzyme A reductase (HMG‐CoA reductase), which converts HMG‐CoA to mevalonic acid, was inhibited by *C. sativum* leading to a decreased production of cell‐associated cholesterol. Lecithin–cholesterol acyltransferase (LCAT) is an enzyme involved in reverse cholesterol transport. Reverse cholesterol transport is a metabolic pathway in which excess cholesterol is transferred from the body's peripheral tissues to the liver to be eliminated. This enzyme is responsible for the transfer of cholesteryl ester to HDL. Coriander has enhanced LCAT and tissue lipase activities leading to more lipid breakdown (Figure 1) (Dhanapakiam et al., 2008).

Coriander seed oil could decrease TC, TG, and LDL and increase HDL in rats (Ramadan et al., 2008). Dry seed powder has reduced cholesterol concentration in the liver, intestine, and proximal and distal colon, decreased cholesterol to phospholipid ratio, and lowered the number of tumors in intestine and colon of rats with colon cancer (Chithra & Leelamma, 2000).

#### 6.9.2 Hypotensive effect

The essential target of hypertension therapy is to manage related diseases, such as heart attack, stroke, and heart failure (HF) (Wolf‐Maier et al., 2004). Coriander has exerted diuretic effects by a similar mechanism to that of furosemide and nonspecific interaction with muscarinic receptors of endothelial cells (Figure 1) (Jabeen et al., 2009). Methanol and aqueous extracts of coriander leaves have indicated increased sodium excretion in the urine more than potassium, which may be a safe profile for diuretic effect (Thuraisingam et al., 2019). This can be considered as a potential hypotensive activity.

#### 6.9.3 Hypoglycemic effect

**FIGURE 2:**
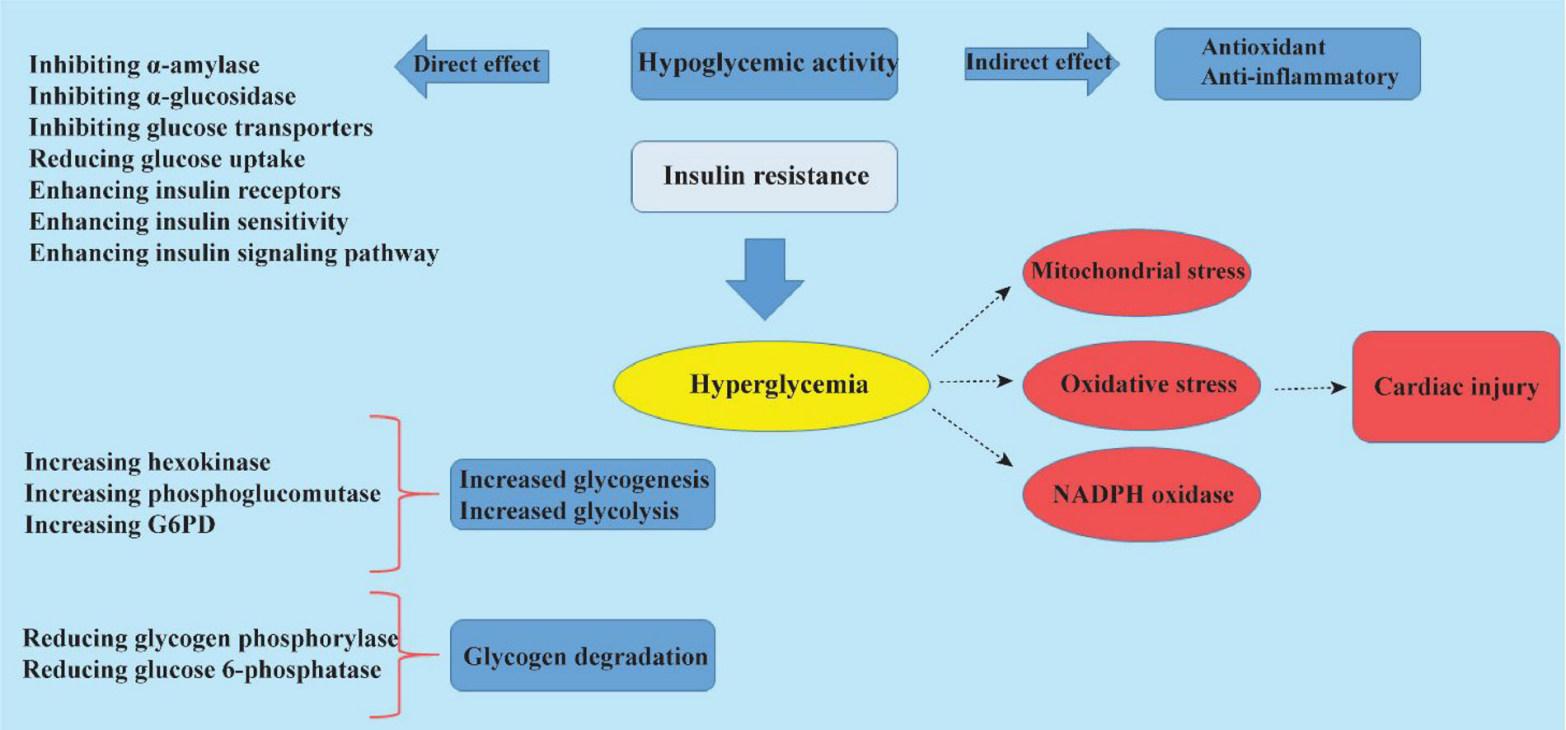
Schematic description of hypoglycemic mechanisms of *C. sativum*

Coriander affected carbohydrate metabolic pathways and resulted in increased glucose consumption in the body. This plant has increased the activities of glucose‐6‐phosphate dehydrogenase, hexokinase, and phosphoglucomutase and enhanced glycogenesis and glycolysis. Coriander can decrease the activity of glycogen phosphorylase and glucose‐6‐phosphatase, leading to a reduction in glycogenolysis and gluconeogenesis (Chithra & Leelamma, 1999). It has improved insulin sensitivity, inhibited α‐amylase and α‐glucosidase activity, prevented glucose transport and absorption, and increased glucose uptake in liver and peripheral tissues (Figure 2) (Aissaoui et al., 2011; Eidi et al., 2009; Gallagher et al., 2003). An in vivo study indicated that treating diabetic mice with a polyphenol fraction of coriander seeds could manage high fasting blood glucose and showed a good hypoglycemic activity (Mechchate et al., 2021).

### 6.10 Anti‐inflammatory effect

Non‐steroidal anti‐inflammatory drugs (NSAIDs) are among the most extensively used drugs that affirm their situation in the Model List of Essential Medicines of WHO. The 2016 Global Burden of Disease results showed that NSAID use is obviously inevitable with expanding musculoskeletal health problems. NSAIDs are additionally reported to provide defense against cancer and heart attacks, beside their analgesic, anti‐inflammatory, and antipyretic potency. In spite of this, the side effects of NSAIDs on respiratory, cardiovascular, hepatic, renal, cerebral, and pulmonary diseases are distressingly demonstrated by evidence from several placebo‐controlled trials. Therefore, there is an expanding need for healthier anti‐inflammatory medications (Bindu et al., 2020). Some plant bioactive compounds like flavonoids or polyphenols have exerted anti‐inflammatory properties through different molecular targets (Nunes et al., 2020). It has been shown that pretreating rats with essential oil and ethanol extract of coriander fruits could relieve ulcer severity and zone in acetic acid‐induced colitis (Heidari et al., 2016). (7α,8α)−3α‐hydroxyl‐12,13α‐dimethyl‐5(6)‐en‐bicyclo[5,3,0]caprolactone isolated from coriander seeds has indicated a remarkable anti‐inflammatory activity by inhibiting NO with an IC_50_ value of 6.25 µM. It has reduced reactive oxygen species (ROS), IL‐6, and tumor necrosis factor alpha (TNF‐α), and expression of inflammatory cytokines like inducible nitric oxide synthase (iNOS) and COX‐2 (Yuan et al., 2020). The antiarthritic impact of essential oil of coriander seeds in rats indicated a substantial decrease in joint diameter, paw volume, and TNF‐α levels (Deepa et al., 2020). In another study, the anti‐inflammatory effect of a traditional Sri Lankan concoction of *C. sativum* and *Coscinium fenestratum* (Gaertn.) Colebr. was studied The results have shown a remarkable decrease in ROS, NO, and iNOS levels in rats. It could also enhance membrane stability, which is another anti‐inflammatory mechanism of the concoction (Kothalawala et al., 2020).

### 6.11 Hepatoprotective effect

Oral administration of ethanol extract of aerial parts to rats with CCL_4_‐induced hepatotoxicity has decreased aspartate transaminase (AST) and alanine transaminase (ALT), increased hepatic antioxidant enzymes like SOD, CAT, and GPX, regenerated hepatocytes, and normalized fatty changes and necrosis of the liver (Sreelatha et al., 2009). Ethanol extract of coriander leaves decreased liver weight, liver enzymes, alkaline phosphatase (ALP), and direct bilirubin in rats with hepatotoxicity. It could exclude fat deposits and deteriorate hepatic necrosis (Pandey et al., 2011). Also, coriander extract has decreased TNF‐α, nuclear factor kappa‐light‐chain‐enhancer of activated B cells (NF‐κB), caspase 3, and necrosis in hepatic ischemia reperfusion injury (Kükner et al., 2021).

### 6.12 Cardioprotective effect

The cardioprotective activity of *C. sativum* extract was assessed in isoproterenol‐induced HF in Wistar rats. Preventive and prophylactic treatment with coriander extract could significantly increase hemodynamic factors, left ventricular activities, and susceptibility to baroreflex. It also suppressed lipid peroxidation, ameliorated lipid profile, and decreased endothelin receptor expression, thus offering substantial defense against HF (Dhyani et al., 2020). Oral administration of methanol extract of coriander seeds has prevented myocardial infarction by suppressing myofibrillar damage. It could decrease creatine kinase‐MB (CK‐MB), LDH, TG, LDL, and VLDL levels and increase HDL (Patel et al., 2012).

### 6.13 Skin disorders

Plants play an important role in the industrial production of perfumes and essential oils in cosmetics (Zhang et al., 2006). In an in vitro study, the antimicrobial effect of green synthesized silver nanoparticles of coriander leaf extract was investigated against *Propionibacterium acnes* and *Malassezia furfur*. The minimum inhibitory concentration (MIC) was 3.1 µg/ml for *P. acnes* and 25 µg/ml for *M. furfur* (responsible for dandruff) (Sathishkumar et al., 2016). Topical administration of the aqueous extract of coriander inhibited the growth of *P. acne* with an MIC of 1.7 mg/ml and *Staphylococcus epidermidis* with an MIC of 2.1 mg/ml (Vats & Sharma, 2012). The calming activity of coriander seed oil has been explored and the results showed that activation of allyl isothiocyanate‐induced transient receptor potential ankyrin 1 (TRPA1) was controlled in keratinocytes–neurons coculture; thus, it can have soothing effects on sensitive skin (Kern et al., 2020).

Photo‐aging is caused by prolonged exposure to sunlight, especially UVA. It is impossible to protect sunlight and it is the main cause of photo‐aging; however, prescribing a suitable sunscreen is beneficial to prevent the photo‐aging process. Huang et al. reported that treating normal human dermal fibroblasts (NHDF) with an ethanol extract of coriander leaves increased procollagen type I and decreased matrix metalloproteinase‐1 (MMP‐1) after UVB irradiation. Also, in vivo results indicated an enhanced epidermal thickness and collagen density (Hwang et al., 2014).

Contact dermatitis is defined as inflammatory responses that occur in the skin as a result of contact with external factors. Coriander has demonstrated a protective effect against contact dermatitis‐like skin lesions induced by 2,4‐dinitrochlorobenzene. Topical administration of coriander extract to dorsal skin prevented lesion development and reduced interferon‐γ (IFN‐γ), immunoglobulin E (IgE), TNF‐α, IL‐1, IL‐4, and IL‐13. Therefore, coriander extract can inhibit lesion development in mice and may be effective as an alternative therapy for contact dermatitis (Park et al., 2014).

## 7 CLINICAL STUDIES

A clinical study conducted on 50 volunteers with type 2 diabetes showed that oral consumption of coriander fruit powder capsules twice daily for 6 weeks resulted in a significant reduction in plasma glucose, TC, TG, and LDL (*p* < 0.001). Therefore, coriander fruit consumption can ameliorate metabolic syndrome and protect against cardiovascular diseases in type 2 diabetic patients (Parsaeyan, 2012). Hypoglycemic effect of powder and aqueous and alcohol extracts of coriander fruits (at doses 2.5 and 4.5 g three times daily for 14 days) has been studied as well. All the tested samples could decrease the blood glucose level and eliminated glycosuria (Waheed et al., 2006).

The results of a monocenter, randomized, placebo‐controlled double‐blind study indicated anti‐inflammatory properties of a topical lotion containing coriander essential oil at concentrations of 0.5% and 1%. The lotion at concentration 0.5% could remarkably reduce UV‐induced erythema. The effectiveness of hydrocortisone (1%) as positive control was greater than this lotion (Reuter et al., 2008). In a nonrandomized clinical trial, topical administration of a cream containing coriander extract was prescribed for diaper dermatitis. The efficacy of this cream was not significant in comparison to hydrocortisone 1% ointment; however, coriander cream can be used for treating mild irritations (Dastgheib et al., 2017). Further, topical administration of a product containing 6% coriander oil exhibited antifungal activity and improved clinical symptoms of fungal infection during the treatment period (Beikert et al., 2013).

Garlic and coriander possess hypotensive activity and alleviate blood lipid profile. Eighty patients were divided into four groups and consumed garlic powder, coriander fruit powder, and a mixture of garlic and coriander fruit powder for 60 days. It was found that oral prescription of garlic and coriander fruit powder (at a dose of 2 g/days) had a vital role in ameliorating metabolic syndrome. Coriander fruit powder decreased TG and its mixture with garlic powder controlled systolic blood pressure (Zeb et al., 2018).

Kumar et al. investigated the effect of coriander fruit syrup on migraines. Patients were selected based on the diagnostic criteria components of International Headache Society. Volunteers took 15 ml of syrup twice daily along with 500 mg/day of sodium valproate for 30 days. The results indicated decreased severity, frequency, and duration of migraine attacks (Kumar & Sinha, 2017).

## 8 SAFETY

Roasted coriander seeds and seeds powder are favorite spices in Iran and India. Coriander fresh leaves are also used in various Iranian cuisine (Burdock & Carabin, 2009). Coriander essential oil was authorized as a harmless dish‐seasoning substance by US Food and Drug Administration (FDA) and Flavor and Extract Manufacturers Association (FEMA) (Burdock & Carabin, 2009). The American Plant Products Association describes coriander fruit as a class I herb that can be used without concern (Gardner & McGuffin, 2013; McGuffin, 1997). However, there have been reports of itching and stinging in lips and mouth (Niinimäki et al., 1995) and rare cases of intensive anaphylactic reaction after coriander consumption (Moneret‐Vautrin et al., 2002).

## 9 FUTURE PERSPECTIVES

Several preparations of coriander have exhibited beneficial effects in treating pain, headache, seizure, migraine, acne, metabolic disorders, parasitic infections, and cancer. Most of these effects are usually attributed to polyphenols and linalool. Polyphenolic compounds of coriander extract can reduce oxidative stress and DNA damage (Cai et al., 2004; Hashim et al., 2005; Tang et al., 2013) by increasing SOD, CAT, and GSH levels and decreasing lipid peroxidation (Samojlik et al., 2010). This can make polyphenols as potential drug candidates for treating metabolic syndrome.

Inhalation of coriander essential oil can improve memory and reduce stress and depression in Alzheimer's disease (Cioanca et al., 2014), which may be due to the antioxidant property of linalool as the major compound of essential oil. The anti‐inflammatory effects of linalool can help to ameliorate Alzheimer's as well since inflammation is a crucial factor in developing this disease. Linalool can prevent glutamate uptake and NO formation and inhibit NMDA receptors and this helps reduce chronic inflammatory pains (Batista et al., 2010). Therefore, coriander and linalool can be considered as potential drug candidates for treating Alzheimer's or other inflammatory conditions, especially neural and CNS diseases.

## 10 CONCLUSION

Coriander as an ancient edible herb has a long tradition. In ITM, coriander is used for healing inflammatory conditions like stomatitis, blepharitis, and hot inflammation of the skin. Based on traditional applications of coriander and different pharmacological and biological activities of the main compounds, it can be concluded that coriander contains many antioxidant components that result in its remarkable antioxidant properties. This can clearly lead to various protective and therapeutic effects that may be beneficial in the food industry and medical treatments. Although the safety of coriander has been confirmed by the United States Food and Drug Administration (FDA) and the Flavor and Extract Manufacturers Association (FEMA), further studies are recommended to investigate the toxicity and adverse effects of this plant when consumed at higher doses and longer periods of time. We suggest herbal pharmaceutical, food, and cosmetics industries to work on formulating oral and topical dosage forms from different parts of coriander and study their effects on skin, gastrointestinal, respiratory, inflammatory, and metabolic diseases.

## AUTHOR CONTRIBUTIONS

**Zahra Sobhani**: Investigation. **Leila Mohtashami**: Investigation. **Mohammad Sadegh Amiri**: Investigation. **Mahin Ramezani**: Investigation. **Seyed Ahmad Emami**: Investigation. **Jesus Simal‐Gandara**: Investigation.

## CONFLICT OF INTEREST

We wish to confirm that this research has not received any specific grant that could have influenced its outcome.
